# Effects of Adsorption and Desorption of Low-Boiling-Point Total Hydrocarbon Gases on Activated Carbon

**DOI:** 10.3390/ma17020384

**Published:** 2024-01-12

**Authors:** Hye-Jin Lee, Jung-Eun Park, Bum-Ui Hong

**Affiliations:** Center for Bio Resource, Institute for Advanced Engineering, Yongin-si 17180, Republic of Korea; hjlee@iae.re.kr (H.-J.L.); jepark0123@gmail.com (J.-E.P.)

**Keywords:** low-boiling-point THCs gas, activated carbon, surface area, pore volume, petrochemical storage

## Abstract

In this study, we selected materials that efficiently adsorb total hydrocarbons (THCs) from petrochemical storage facilities and applied four types of activated carbons to adsorb THCs to evaluate their properties. Four gases with low boiling points, namely, ethylene, ethane, propylene, and propane, generated via petrochemical storage facilities, were selected and mixed to a constant concentration with four types of materials and used to investigate the adsorption capacities and desorption properties. The adsorbents comprised two raw materials and two chemically activated materials. The specific surface areas of activated palm (2085 m^2^/g) and coal (1752 m^2^/g), which are chemically activated carbons, exhibited a twofold increase compared to those of raw palm (1232 m^2^/g) and coal (946 m^2^/g). Thus, we identified the correlations between the physical properties of the activated carbon adsorption materials and their adsorption capacities for four low-boiling-point THCs generated by petrochemical storage facilities.

## 1. Introduction

The size of the global petrochemical market was estimated at USD 617 trillion in 2023 and is projected to increase at a 6% compound annual growth rate to USD 1042 billion in 2032 [1]. The global market size of polyethylene and polypropylene, which are fundamental materials in the plastic manufacturing process, is expected to grow from 113 and 82 million tons in 2023 to 135 and 105 million tons in 2030, respectively [2,3].

In 1983, the U.S. Environmental Protection Agency (EPA) established atmospheric pollutant standards for catalyst preparation, polymerization reactor, decanter, and neutralizers; diluent separation and recovery; and drying, extrusion, and pelletizing facilities [4]. However, efforts to monitor the air pollutants generated by storage facilities for products synthesized using these processes remain negligible.

A variety of hydrocarbons ranging between C_2_ and C_18_ are produced in the product storage facilities of petrochemical plants, and propylene and ethylene account for the largest and second-largest emissions of THC in petrochemical plants, respectively [5,6]. These two gases are used as monomers or comonomers in the plastic production process, and a similar trend may be expected for the storage facilities of the products produced.

In South Korea, emission allowances for solid storage facilities in petrochemical plants should be reduced to <200 ppm by 2025. In petrochemical product storage facilities, total hydrocarbons (THCs) are generated at an average level of 100–2000 ppm, and technologies for processing them are required [7]. Therefore, new techniques are needed to process gases with a low boiling point; however, processing the low concentration of low-boiling-point THCs in the large volume of air generated from petrochemical storage facilities is challenging.

A removal process including a wet scrubber, open combustion through a flare stack, and adsorption using adsorbents to control the THCs generated by petrochemical plants is typically applied [8]. Among these, techniques applying a wet scrubber are challenging owing to the insolubility of THC. Furthermore, blowers are used to inject a large amount of outside air for degassing to reduce the concentration of THCs in the storage facility. There is some risk of explosion during the application of the flare system, and there are concerns that the flare stack can mar the surrounding environment. Moreover, these systems require a great deal of energy to operate. Thus, adsorption systems are among the most suitable techniques for dealing with THC gases with low boiling points, such as ethylene and propylene [9].

Several studies have been conducted on zeolite and activated carbon as ethylene and propylene adsorbents [10,11,12], which increased the pressure to 100 bar or reduced the temperature to −100 °C to adsorb low-boiling-point THCs, which exhibit a boiling point of −100 °C or lower. Therefore, mild adsorption conditions need to be developed because these conditions are difficult to use in industrial sites. Golipour et al. compared four types of zeolites to adsorb ethylene gas and found that the total pore volume of zeolite remarkably affected the adsorption capacity, whereas the specific surface area did not have a considerable influence on the adsorption capacity [13]. Although zeolite has been employed in several studies on adsorption [14,15,16], Schmittmann et al. compared zeolite and activated carbon for ethylene and propylene adsorption. The capacity of zeolite (approximately 2 mol/kg for ethylene and 2 mol/kg for propylene) was lower than that of activated carbon (approximately 6 mol/kg for ethylene and 8 mol/for propylene), although it is more expensive [17,18,19]. Generally, the price of zeolite 13X (12 $/kg) is higher than that of activated carbon. Especially, the price varies depending on the quality of activated carbon, and as the specific surface area increases below 1000 m^2^/g, 1500 m^2^/g, and 2000 m^2^/g, the price increases to 1.4 $/kg, 8 $/kg, and 10 $/kg, respectively [18]. Alkene with a quadripolar moment formed a strong cation−π interaction with sodium ions on the zeolite surface and exhibited a larger adsorption quantity than alkane with a neutral charge distribution. However, when activated carbon was used, the adsorption capacity for ethylene and propylene increased two- to three-fold compared to that when zeolite was used because the specific surface area of activated carbon is higher than that of zeolite (activated carbon: 1600 m^2^/g, zeolite: 602 m^2^/g) [17]. Research on activated carbon is essential because it is more beneficial for the adsorption of low-boiling-point THC than zeolite [20,21,22]. Ye et al. studied the ability of 15 types of activated carbons to adsorb ethylene and propylene [23]. They reported that an increase in the specific surface area of activated carbon increased the total adsorption capacity of ethylene and propylene because the larger volume of micropores corresponded to a higher adsorption capacity, especially for ethylene. However, the adsorption capacity, including the effective adsorption capacity, was mainly proportional to the concentrations of the initial feed. Therefore, the major factors affecting the adsorption of low-boiling-point gases have thus far remained unclear.

Therefore, in this study, we considered methods for identifying materials suitable for the adsorption of low-concentration THC from large air volumes in petrochemical storage facilities and described their properties. To this end, activated carbons with different specific surface areas were selected as adsorbents. Additionally, we compared the properties of activated carbons suitable for the adsorption of four gases, including ethylene, ethane, propylene, and propane.

## 2. Materials and Methods

### 2.1. Materials

To simulate the THCs generated in a petrochemical plant, low-boiling-point ethylene, ethane, propylene, and propane mixed gases were purchased and used in an experimental evaluation (JC GAS, Anseong, Republic of Korea). The total concentration of the low-boiling-point mixed gases was 1000 ppm. Ethylene, ethane, propylene, and propane were manufactured to 926, 28, 18, and 28 ppm, respectively (N_2_ balance). The basis for calculating the fraction and properties of low-boiling-point THC gases is shown in Table 1.

Hexane gas (JC GAS, Anseong, Republic of Korea) was also purchased and used as a THC with a high boiling point. Granular palm- and coal-based activated carbons with 3–4 mm particle sizes were also purchased and used (palm-based activated carbon: DAELIM, Seoul, Korea; carbon-based active coal: CS Corporation, Seongnam, Republic of Korea). They were dried at 105 °C for >24 h and cooled in a desiccator with silica gel at 25 °C before the experiment.

### 2.2. Experimental Instruments and Procedures

#### 2.2.1. Chemical Activation

To compare the adsorbent, four types of activated carbon were purchased as adsorbents and upgraded via chemical activation. The purity of KOH (SAMCHEON CHEMICAL, Ansan, Republic of Korea) as a chemical activator was 95%, and it was used as described below. The ratio of precursor to raw materials and activator for KOH was 1:3, and the solid mixture was placed in a horizontal reactor. Nitrogen was released at 1 L/min, heated at 10 °C/min to 850 °C, and subsequently activated for 3 h. The resulting samples were washed using distilled water at pH 7 and dried for >24 h at 105 °C before being used in the adsorption experiments. The palm-based activated carbon, coal-based activated carbon, chemically activated palm-based activated carbon, and activated coal are denoted as R_P (raw palm), R_C (raw coal), A_P (activated palm), and A_C (activated coal), respectively.

#### 2.2.2. Analysis of Properties of Adsorbents

The specific surface area and geometric structural properties of activated carbon were examined via nitrogen adsorption and desorption at −196 °C using a TriStar II 3020 (Micromeritics, Norcross, GA, USA). The specific surface area was determined using the Brunauer–Emmett–Teller (BET) method, and the characteristics of the pore structure were evaluated using the Barrett–Joyner–Halenda (BJH) method [24].

X-ray photoelectron spectroscopy (XPS) was performed using a PHI 5000 VersaProbe (Ulvac-PHI, Hagisono, Japan) to determine the state of surface oxygen on the surface of activated carbon following the chemical activation process. The XPS was calibrated using Al Kα (biding energy = 1486.6 eV) for the radiation source and the C1s peak (284.6 eV) for the spectrum [25,26]. Raman spectroscopy was also performed using a LabRam ARAMIS IR2 (HORIBA Jobin Yvon, Glasgow, UK) to investigate the defects on the surface of the activated carbon [27]. Scanning electron micrographs of the adsorbent were recorded using a JSM-7800F Prime scanning electron microscope (JEOL Ltd., Tokyo, Japan).

#### 2.2.3. Adsorption and Desorption Experiments

Figure S1 shows a schematic illustration of the adsorption and desorption experiments. A quartz reactor 3 cm in diameter and 20 cm in height was used, and a bed was installed inside the reactor and filled with activated carbon. For the adsorption experiment, 5 g of activated carbon was placed in the petrochemical reactor under a flow of 100 mL/min of THCs under 100 ppm total feed concentration conditions. The space velocity depended on the density of activated carbon and varied in the range of 0.014–0.158 s^−1^. Real-time quantitative analysis of the gas that passed through the adsorbent was conducted using a Polaris flame ionization detector (FID, Pollution Analytical Equipment, Budrio, Italy). The odor threshold and irritation level of THC are at the level of parts per billion, and the total adsorption capacity of an adsorbent is difficult to use as a design factor for equipment that removes exhaust THC gases from petrochemical storage facilities. Therefore, we detected and calculated the effective adsorption capacity for less than 1 ppm at the breakthrough point [28,29,30].

The adsorption experiment was stopped when effective adsorption was reached, and the effective desorption reaction was then started immediately. The desorption reaction was implemented using the same reactor and methods. The adsorption and desorption reactions were performed at room temperature (25 °C) and atmospheric pressure. A quantitative analysis of the gas that passed through the reactor was performed using the Polaris FID by releasing nitrogen at room temperature at a rate of 100 mL/min.

## 3. Results and Discussion

### 3.1. Properties of Activated Carbons

Generally, the specific surface area and pore properties of the adsorbent affected the adsorption capacity dramatically [9]. Therefore, we wanted to change the specific surface area and pore properties of the adsorbents with activation, as listed in Table 2. The coal-based activated carbon (R_C) and palm-based activated carbon (R-P) exhibited substantial increases in specific surface area and pore volume, and the micropore volume increased by a factor of >2 after chemical activation. The external pore volume of the R_P increased by a factor of >3, in contrast to the constant volume of external pores (<2 nm in size) for the R_C.

**Figure 1:**
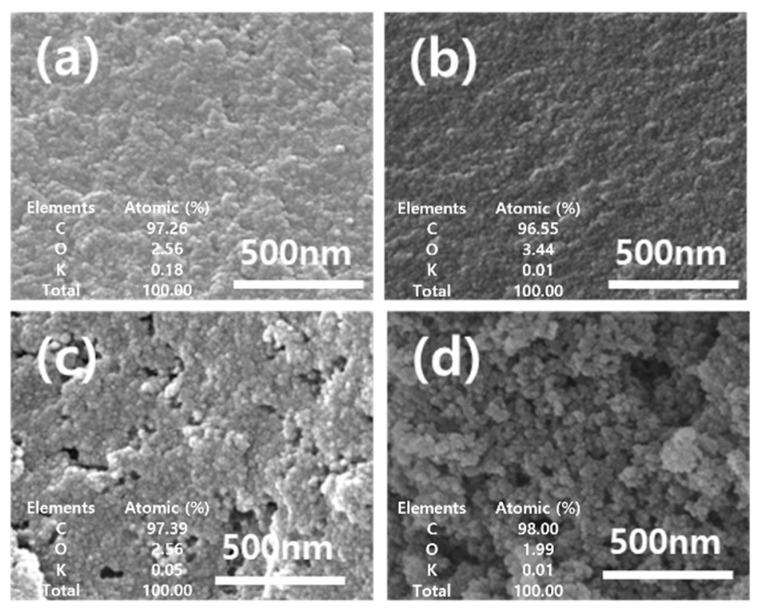
SEM images and the element ratio with EDS analysis of (**a**) R_P, (**b**) R_C, (**c**) A_P, and (**d**) A_C.

An analysis using scanning electron microscopy showed that carbon texture and the development of porosity were affected by the characteristics of chemical activation, as shown in Figure 1. The R_P and R_C had similar morphologies with smooth surfaces without pores. After activation, A_P and A_C yielded rougher textures with heterogeneous surfaces and an increased randomly distributed pore size. A comparison between raw materials and activated materials at the activation showed their surface characteristics. Also, we analyzed the atomics of carbon (C), oxygen (O), and potassium (K) on surface-activated carbons as adsorbents using EDS. The elements and atomics at the surface are depicted in Figure 1. From the results, the carbon ratios of R_P and R_C were 97.26 and 96.55, respectively. After chemical activation, the carbon ratio of A_P and A_C was increased by increasing the carbon density. The inorganic component, potassium, was decreased by the washing process after chemical activation.

**Figure 2:**
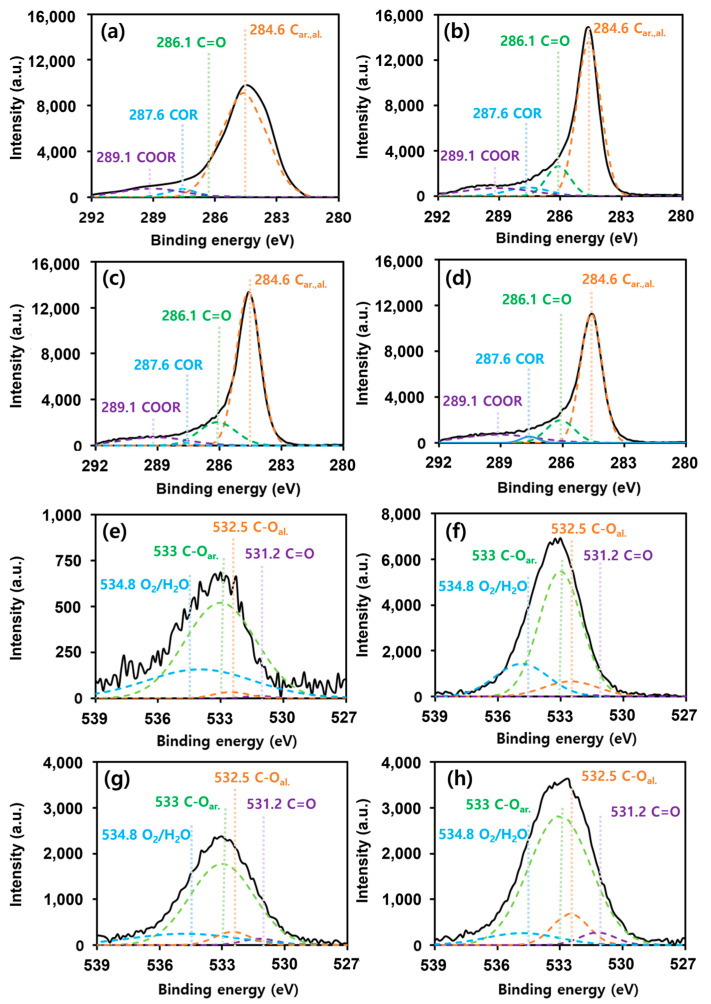
High-resolution XPS spectra. C1s XPS spectra of (**a**) R_P, (**b**) A_P, (**c**) R_C, and (**d**) A_C. O1s XPS spectra of (**e**) R_P, (**f**) A_P, (**g**) R_C, and (**h**) A_C.

The XPS and Raman spectra were used to examine the changes in the surface oxygen groups following activation and to determine the correlation with the adsorption capacity. We performed an XPS analysis to assess the oxidation states of carbon and oxygen on the activated carbon surface and how they changed to determine the polarity of the surface according to the activation process. Figure 2 shows the C1s and O1s XPS spectra of the four types of activated carbons. The 289.1, 286.1, and 287.6 eV peaks correspond to O-C=O, C=O, and C-O, respectively [31]. By fitting the peak of O1s with three oxygen species, we confirmed that after activation of both the palm- and coal-based activated carbons, the overall peak area of O1s increased, whereas the peak areas corresponding to O-C=O, C=O, and C-O decreased. This could be attributed to the increased surface oxygen on the activated carbon surface owing to the reaction with the activator KOH during the activation process.

**Figure 3:**
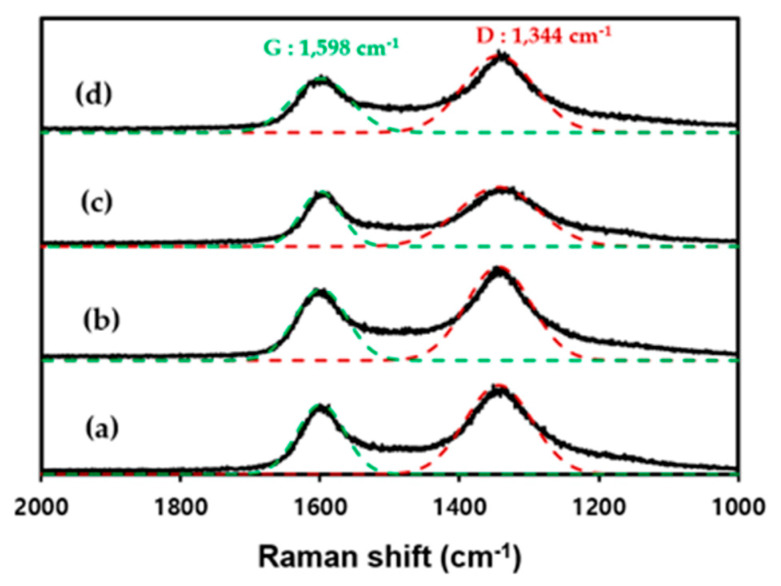
Raman spectra of (**a**) R_P, (**b**) A_P, (**c**) R_C, and (**d**) A_C.

Raman spectroscopy is useful for analyzing the structures of carbon surfaces and can indicate the quantitative changes in the functional groups present on an activated carbon surface. Raman analysis of the four types of activated carbons was performed to determine the changes in the surface of the activated carbon due to chemical activation, as shown in Figure 3. Two normalized peaks based on the G-band (1598 cm^−1^) and D-band (1344 cm^−1^) are evident. The G-band (1598 cm^−1^) corresponds to an aligned graphitic structure, whereas the D-band (1344 cm^−1^) corresponds to amorphous structures and surface defects [27]. A higher intensity ratio of the D- to the G-band (I_D_/I_G_) corresponds to a more disorderly crystal structure. Both R_P and R_C underwent chemical activation, and the I_D_/I_G_ ratio increased from 1.80 to 1.67 and from 2.15 to 1.72, respectively. This result indicated an increase in oxygen on the surface of the activated carbon as the activated carbon underwent the chemical activation process using KOH, which is consistent with the abovementioned XPS results.

### 3.2. Adsorption Characteristics of Activated Carbons

**Figure 4:**
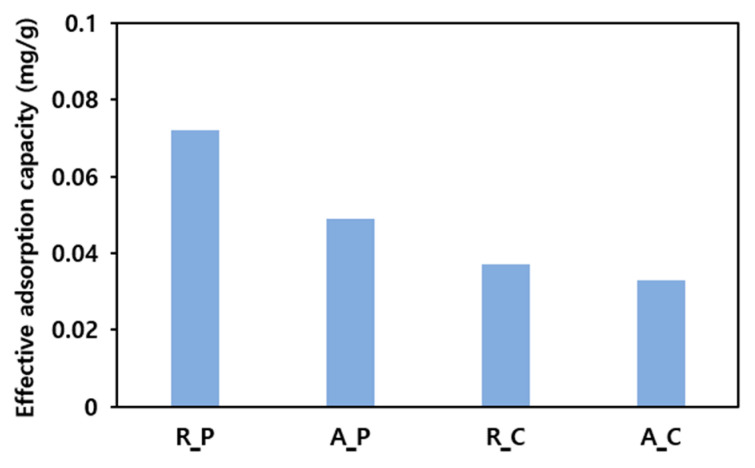
Results of the low-boiling-point gas adsorption experiment (5 g of adsorbent, feeding gas concentration: 100 ppm, flow rate of adsorption: 100 mL/min, temperature of adsorption: 25 °C).

The results of applying the four types of activated carbons to the adsorption of low-boiling-point mixed gases are shown in Figure 4. Regardless of activation, the palm-based activated carbon exhibited a higher adsorption capacity than the coal-based activated carbon. For both the palm- and coal-based activated carbons, the inactivated state was more beneficial for adsorption. Considering that the specific surface area increased after activation, it was observed that the specific area did not affect the adsorption capacity for low-boiling-point THC gases significantly. The adsorption capacity—an inherent property of an adsorbent—is decreased by a reduction in the number of absorption sites or by the promotion of the desorption reaction. Thus, the adsorption capacity of a material can be reduced owing to a decrease in the number of absorption sites or to an acceleration of the desorption reaction due to a change in pore size distribution on the activated carbon surface after the activation process. Therefore, the factors that influence the effective adsorption capacity between these two possibilities should be investigated.

Hydrocarbon adsorption depends on the polarity of the adsorbent surface owing to its structural characteristics. Particularly, for alkenes such as ethylene and propylene, which account for a substantial proportion of the low-boiling-point gases generated in petrochemical storage facilities, the adsorption is dominant on the polarized adsorbent surface [17]. The degree of polarity on the activated carbon surface is affected by the states and quantities of the bonding of surface oxygen functional groups. However, the adsorption capacity was reduced after activation despite the overall increase in the quantity of surface oxygen in this research. Hence, the surface oxygen group was not a major factor in increasing the adsorption capacity.

**Figure 5:**
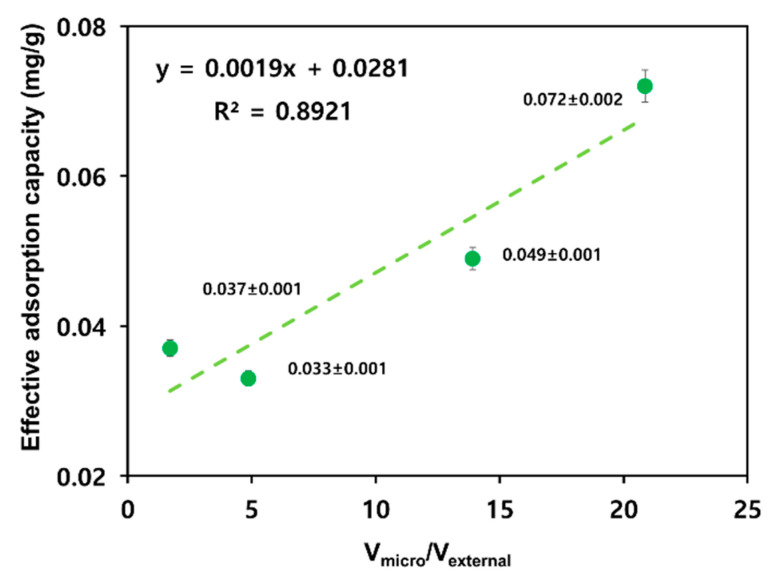
Relationship between the effective adsorption capacity and the micropore volume versus external pore volume.

Figure 5 shows a correlation between the V_micro_/V_external_ ratios on the surfaces of four types of activated carbons and the effective adsorption capacity. Activating both R_C and R_P increased the volume of micropores by a factor >2. A_C exhibited a constant external pore volume, whereas the external pore volume of A_P increased by a factor of >3 (see Table 2). These differences are related to changes in the effective adsorption capacities after R_C and R_P activation. Following activation, R_C exhibited a minor reduction in effective adsorption capacity (0.037 to 0.033 mg/g), whereas R_P exhibited a remarkable decrease of 0.023 mg/g (0.072 to 0.049 mg/g). Therefore, macropores exposed on the external surface and mesopores connected to them serve as channels for the adsorbent, and micropores are the main adsorption sites. Therefore, the V_micro_/V_external_ ratio is essential for adsorption.

As shown in Table 3, we compared the adsorption capacity of values in previous research with those in our research. Without pressure conditions, the total adsorption capacity was mentioned at 0.07 g/g-ethylene and 0.14 g/g-propene under high-concentration feeding conditions [17]. Our total adsorption capacity is lower than in previous research because our experiment conditions were harder than those in previous research in terms of as low feeding concentration and mixed gas conditions. Previous studies did not provide information on the effective absorption capacity, so when the effective adsorption capacity is calculated by applying the same ratio as in our research results, the effective adsorption capacity of ethylene is 0.12 mg/g, and propene is 0.25 mg/g, which is similar to the result of the effective adsorption capacity of the mixed gases.

### 3.3. Desorption Characteristic of Activated Carbons

An adsorbate with a smaller molecular mass is desorbed more easily because less energy is needed for desorption [32]. Therefore, low-boiling-point gases are easily detached, even if they are adsorbed on an active carbon surface; thus, the absorption and desorption reactions occur simultaneously. The desorption rate for the adsorbate gas is calculated using Equation (1) as follows.

(1) $$\mathrm{Desorption}\mathrm{rate}\;(\%)=\frac{\mathrm{Quantity}\mathrm{of}\mathrm{desorbed}\mathrm{THC}\;(g)}{\mathrm{Quantity}\mathrm{of}\mathrm{adsorbed}\mathrm{THC}\;(g)}\;\times \;100$$

**Figure 6:**
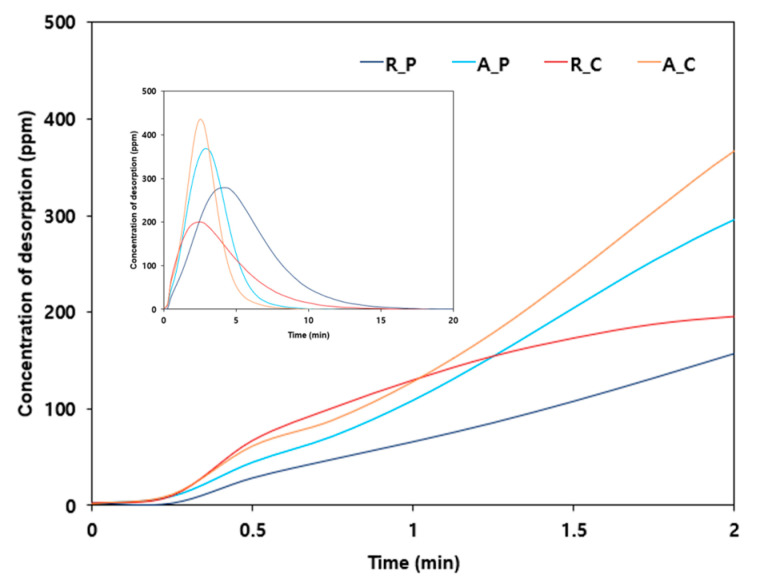
Desorption curve of low-boiling-point mixed gases for the four types of adsorbents (adsorbent: 5 g of R_P; feeding gas concentration: 100 ppm, flow rate of adsorption and desorption: 100 mL/min, temperature of adsorption and desorption: 25 °C). Main graph: low range (<2 min); inset graph: all data.

To confirm the reduction in adsorption capacity due to the accelerated desorption reaction, the low-boiling-point gases with a concentration of 100 ppm were released at 100 mL/min into the four types of activated carbon. Then, at the breakthrough time after effective adsorption, a gas was released at 1 L/min at room temperature for a desorption experiment. As shown in Figure 6, R_P with the highest V_micro_/V_external_ ratio (20.862) had the lowest slope of desorption, as expected, and the R_C with the lowest V_micro_/V_external_ ratio (1.708) had the highest slope of desorption. This result clearly indicates the correlation between the distribution properties of pores on activated carbon surfaces and the desorption reaction of low-boiling-point gases. It also clearly shows that a higher V_micro_/V_external_ ratio was more beneficial for the adsorption of low-boiling-point gases. Therefore, to effectively adsorb low-boiling-point gases such as ethylene and propylene, a material that can inhibit the desorption reaction should be applied. Such materials have a high V_micro_/V_external_ ratio for pore size distribution, forming a complex, and a narrow channel shape, through which the adsorbate must pass during the desorption process.

A higher ratio of the micropore volume to the external pore volume on the activated carbon surface (V_micro_/V_external_) makes the desorption reaction more difficult because the already adsorbed gas must be released via a complex and long channel. In contrast, a lower V_micro_/V_external_ corresponds to a larger volume of large-diameter pores on the surface. Even if the adsorbate is adsorbed deeply into the surface of the activated carbon, the desorption reaction proceeds easily and quickly along a broad and simple channel. That is, with a higher V_micro_/V_external_ ratio on the activated carbon surface, the desorption reaction is inhibited to a greater degree, which leads to a higher adsorption capacity.

**Figure 7:**
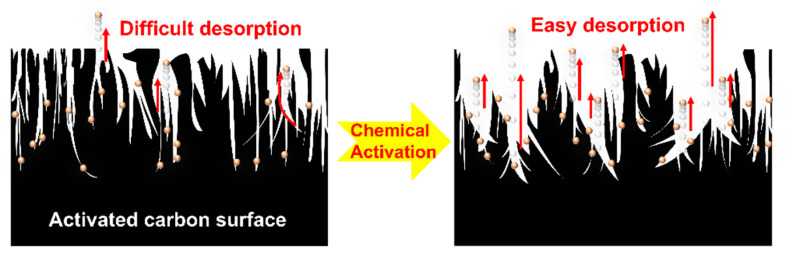
Schematic of the desorption reaction according to the characteristics of pores on the activated carbon surface (dots: adsorbate, arrows: desorption reaction).

The results indicate that activation facilitates the desorption reaction by reducing the adsorption capacity as the desorption channel becomes broader and simpler. To explain that low-boiling-point THCs are dependent on pores, desorption was modeled as shown in Figure 7. A higher V_micro_/V_external_ ratio corresponds to a higher adsorption capacity; it indicates the correlation between the pore size distribution of the activated carbon surface and the effective absorption capacity of the low-boiling-point gases. The low-boiling-point gases were strongly desorbed with adsorption, and the adsorption capacity may have decreased owing to changes in surface properties that promoted desorption reactions during the activation process.

### 3.4. Effect of the Feeding Gases

Among the adsorbents, the R_P showed the highest effective adsorption capacity for four types of low-boiling-point THC gases in this research. In the field condition, the THC gases were mixed using a low boiling point and a high boiling point. The experiment described here was conducted under different feeding conditions as follows: (1) 100 ppm of methane (b.p. −162 °C), (2) 100 ppm of low-boiling-point THCs (ethylene b.p. −103.7 °C, ethane b.p. −89 °C, propene b.p. −47.6 °C, propane b.p. −42 °C), and (3) mixed THCs including 30 ppm of hexane (b.p. 69 °C) and 70 ppm of low-boiling-point THCs on R_P under 100 mL/min.

**Figure 8:**
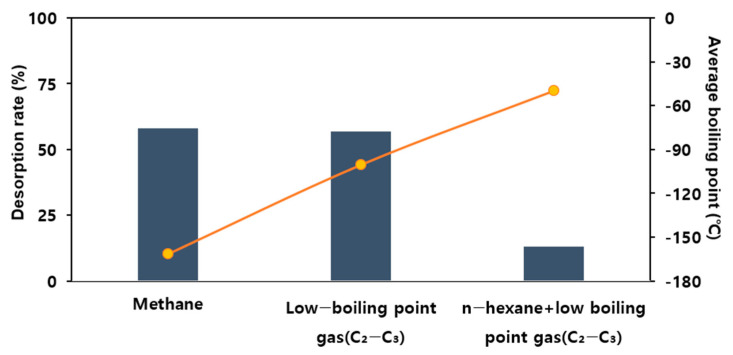
Desorption rate for the adsorbate (adsorbent: 5 g of R_P, feeding gas total concentration 100 ppm, flow rate: 100 mL/min, temperature: 25 °C).

As shown in Figure 8, the effective adsorption capacities of methane, low-boiling-point THCs, and mixed THCs were 0.0462 mg/g, 0.072 mg/g, and 2.884 mg/g, respectively. These results indicate that the boiling point is inversely proportional to the effective adsorption capacity.

Adsorbing 100 ppm methane gas, which has the lowest boiling point among the THCs, and desorbing it under the same conditions resulted in a 58% desorption rate. Releasing low-boiling-point mixed gases at 100 ppm and then desorbing them under the same conditions resulted in a 57% desorption rate. Adsorbing a relatively high-boiling-point gas mixed with 30 ppm hexane and 70 ppm low-boiling-point gases and then releasing nitrogen at room temperature resulted in a 13% desorption rate. Therefore, boiling-point gases with a smaller molecular mass were easily adsorbed and desorbed. Thus, the facilitation of the adsorption reaction and inhibition of the desorption reaction are required to adsorb low-boiling-point gases efficiently.

## 4. Conclusions

We investigated the properties of materials that can effectively absorb low-boiling-point gases, namely, ethylene, ethanol, propylene, and propane, which are generated in petrochemical product storage facilities. The low-boiling-point gases have an extremely low ad/desorption energy; therefore, the adsorption and desorption reactions occur simultaneously. To increase the adsorption capacity of low-boiling-point THCs, it is necessary to select an adsorbent that has properties that not only promote adsorption but also suppress desorption. Therefore, we compared the adsorption capacities of palm- and coal-based activated carbons and investigated their surface properties before and after chemical activation. We further examined whether surface oxygen on activated carbon could function as an adsorption site in adsorption reactions; however, no correlation was found with the adsorption capacity of low-boiling-point gases. Macropores exposed on the external surface and mesopores connected to them serve as channels for the adsorbent, and micropores are the main adsorption sites. Therefore, the V_micro_/V_external_ ratio is essential for adsorption. We identified an increasing trend in the adsorption capacity with an increasing V_micro_/V_external_ ratio. Among the adsorbents, R_P exhibited the highest V_micro_/V_external_ ratio (20.862) and the highest adsorption capacity for low-boiling-point mixed gases (0.072 mg/g). Owing to its material characteristics, R_P has rich micropores and few external pores on the surface. Therefore, adsorbed low-boiling-point gases must be desorbed via a complex and long channel, which inhibits the desorption reaction and increases the adsorption capacity. These results are consistent with those of previously published reports showing that the adsorption capacity depends on the distribution of pore sizes instead of the specific surface area of the porous carbon. In summary, the adsorption capacity of low-boiling-point gases is predominantly affected by the V_micro_/V_external_ ratio rather than the specific surface area of the adsorbent.

## Figures and Tables

**Table 1:** The fraction of low-boiling-point THC gases from petrochemical storage plants in South Korea and gas properties.

Low- Boiling-Point THC Gases	Gas Formular	Molar Mass (g/mol)	Density (kg/m^3^)	Boiling Point (°C)	Detected Gas Conc. (ppm)	Fraction (%)
Ethylene	C_2_H_6_	30.07	1.35 at 0 °C	−88.5	66.1	92.6
Ethane	C_2_H_4_	28.05	1.17 at 15 °C	−103.7	2.0	2.8
Propylene	C_3_H_6_	42.08	1.81 at 15 °C	−47.6	1.3	1.8
Propane	C_3_H_8_	44.09	2.00 at 0 °C	−42.3	2.0	2.8
Total					71.4	100.0

**Table 2:** Physical properties of the four types of activated carbon.

	R_P	A_P	R_C	A_C
Specific surface area (m^2^/g)	1232	2085	946	1752
V_total_ (cm^3^/g)	0.504	1.033	0.451	1.018
V_micro_ (cm^3^/g)	0.481	0.964	0.285	0.845
V_external_ (cm^3^/g)	0.023	0.069	0.167	0.174
V_micro_/V_external_ (-)	20.862	13.909	1.708	4.864

**Table 3:** The effective adsorption capacity in previous research and our research.

	Feed Gas	Concentration (ppm)	Flow Rate (mL/min)	Effective Adsorption Capacity (mg/g)	Total Adsorption Capacity (g/g)
Our results	Ethylene + Ethane +Propylene +Propane	100	100	0.07	0.04
Previous research [17]	ethylene	990,000	200	-	0.07
	propene	990,000	200	-	0.14

## Data Availability

Data are contained within the article.

## Supplementary Materials

The following supporting information can be downloaded at: https://www.mdpi.com/article/10.3390/ma17020384/s1. Figure S1. Schematic of the adsorption and desorption experiments.

## References

[1] Petrochemical Market Size to Worth Around US$ 1041.79 Bn by 2032. https://www.precedenceresearch.com/petrochemical-market.

[2] Market Volume of Polyethylene Worldwide from 2015 to 2022, with a Forecast for 2023 to 2030. https://www.statista.com/statistics/1245162/polyethylene-market-volume-worldwide/.

[3] Market Volume of Polypropylene Worldwide from 2015 to 2022, with a Forecast for 2023 to 2030. https://www.statista.com/statistics/1245169/polypropylene-market-volume-worldwide/.

[4] Environment Protection Agency (EPA) (1983). Control of VOCs Emissions from Manufacture of HDPE, PP, and PS Resins(EPA-450).

[5] Baek S.-O., Seo Y.-K., Kim J.-H. (2020). Occurrence and distributions of volatile organic compounds in the ambient air of large petrochemical industrial complexes: Focusing on Daesan area. J. Korean Soc. Atmos. Environ..

[6] Piringer O.G., Baner A.L. (2008). Plastic Packaging Materials for Food: Barrier Function, Mass Transport, Quality Assurance, and Legislation.

[7] YES ENC Inc. (2020). A Study on Improving THC Emission Regulations Relating to PP and PE Storage Silos.

[8] Ramboll Environ (2017). Air Pollution Control Technology Review for the Chemical and Fertilizer Sectors.

[9] Reimerink W.M.T.M., Kleut D. (1999). Air pollution control by adsorption. Studies in Surface Science and Catalysis.

[10] Divekar S., Nanoti A., Dasgupta S., Aarti, Chauhan R., Gupta P., Garg M.O., Singh S.P., Mishra I.M. (2016). Adsorption equilibria of propylene and propane on zeolites and prediction of their binary adsorption with the ideal adsorbed solution theory. J. Chem. Eng. Data.

[11] Andrade M., Parnell A.J., Bernardo G., Mendes A. (2021). Propane selective carbon adsorbents from phenolic resin precursor. Microporous Mesoporous Mater..

[12] Bläker C., Pasel C., Luckas M., Dreisbach F., Bathen D. (2017). Investigation of load-dependent heat of adsorption of alkanes and alkenes on zeolites and activated carbon. Microporous Mesoporous Mater..

[13] Golipour H., Mokhtarani B., Mafi M., Moradi A., Godini H.R. (2020). Experimental measurement for adsorption of ethylene and ethane gases on copper-exchanged zeolites 13X and 5A. J. Chem. Eng. Data.

[14] Mofarahi M., Salehi S.M. (2013). Pure and binary adsorption isotherms of ethylene and ethane on zeolite 5A. Adsorption.

[15] Limtrakul J., Nanok T., Jungsuttiwong S., Khongpracha P., Truong T.N. (2001). Adsorption of unsaturated hydrocarbons on zeolites: The effects of the zeolite framework on adsorption properties of ethylene. Chem. Phys. Lett..

[16] Triebe R.W., Tezel F.H., Khulbe K.C. (1996). Adsorption of methane, ethane and ethylene on molecular sieve zeolites. Gas Sep. Purif..

[17] Schmittmann S., Pasel C., Luckas M., Bathen D. (2020). Adsorption of light alkanes and alkenes on activated carbon and zeolite 13X at low temperatures. J. Chem. Eng..

[18] (2023). Activated Carbon Market Analysis the Untapped Industry Expansion 2023–2030, Market Reports Word. https://www.linkedin.com/pulse/activated-carbon-market-analysis-untapped-industry/.

[19] Babel S., Kurniawan T.A. (2003). Low-cost adsorbents for heavy metals uptake from contaminated water: A review. J. Hazard. Mater..

[20] Costa E., Calleja G., Marron C., Jimenez A., Pau J. (1989). Equilibrium adsorption of methane, ethane, ethylene, and propylene and their mixtures on activated carbon. J. Chem. Eng. Data.

[21] Reich R., Ziegler W.T., Rogers K.A. (1980). Adsorption of methane, ethane, and ethylene gases and their binary and ternary mixtures and carbon dioxide on activated carbon at 212–301 K and pressures to 35 atmospheres. Ind. Eng. Chem. Process Des. Dev..

[22] Mofarahi M., Sadrameli M., Towfighi J. (2003). Characterization of activated carbon by propane and propylene adsorption. J. Chem. Eng. Data.

[23] Ye P., Fang Z., Su B., Xing H., Yang Y., Su Y., Ren Q. (2010). Adsorption of propylene and ethylene on 15 activated carbons. J. Chem. Eng. Data.

[24] Kumar A., Jena H.M. (2015). High surface area microporous activated carbons prepared from Fox nut (*Euryale ferox*) shell by zinc chloride activation. Appl. Surf. Sci..

[25] Zielke U., Hüttinger K.J., Hoffman W.P. (1996). Surface-oxidized carbon fibers: I. Surface structure and chemistry. Carbon.

[26] Smith M., Scudiero L., Espinal J., McEwen J.S., Garcia-Perez M. (2016). Improving the deconvolution and interpretation of XPS spectra from chars by ab initio calculations. Carbon.

[27] Park J.E., Lee G.-H., Choi M., Dar M.A., Shim H.-W., Kim D.-W. (2015). Comparison of catalytic performance of different types of graphene in Li–O_2_ batteries. J. Alloys Compd..

[28] Ruth J.H. (1986). Odor Thresholds and Irritation Levels of Several Chemical Substances: A Review. Am. Ind. Hyg. Assoc. J..

[29] Ferreira D., Magalhaes R., Taveira P., Mendes A. (2011). Effective Adsorption Equilibrium Isotherms and Breakthroughs of Water Vapor and Caron Dioxide on Different Adsorbents. Ind. Eng. Chem. Res..

[30] Park J.E., Jo E.S., Lee G.B., Lee S.E., Hong B.-U. (2023). Adsorption Capacity and Desorption Efficiency of Activated Carbon for Odors from Medical Waste. Molecules.

[31] Liang X., Chi J., Yang Z. (2018). The influence of the functional group on activated carbon for acetone adsorption property by molecular simulation study. Microporous Mesoporous Mater..

[32] Kim A.G. (1974). Low-Temperature Evolution of Hydrocarbon Gases from Coal.

