# Supplementary material for: Effects of Adsorption and Desorption of Low-Boiling-Point Total Hydrocarbon Gases on Activated Carbon
Source: Materials (Basel). 2024 Jan 12;17(2):384. doi: 10.3390/ma17020384 (PMC10820054; doi:10.3390/ma17020384)
Supplement: Supplementary file 1 [file materials-17-00384-s001.zip › materials-2733953-supplementary.pdf]

Supplementary materials

Effects of Adsorption and Desorption of Low-Boiling-Point THC Gases on Activated Carbon

Hye-Jin Lee, Jung-Eun Park and Bum-Ui Hong *

Center for Bio Resource, Institute for Advanced Engineering; Yongin-si 17180, Republic of Korea; hjlee@iae.re.kr (H.-J.L.); jepark0123@gmail.com (J.-E.P.)
* Correspondence: buhong@iae.re.kr

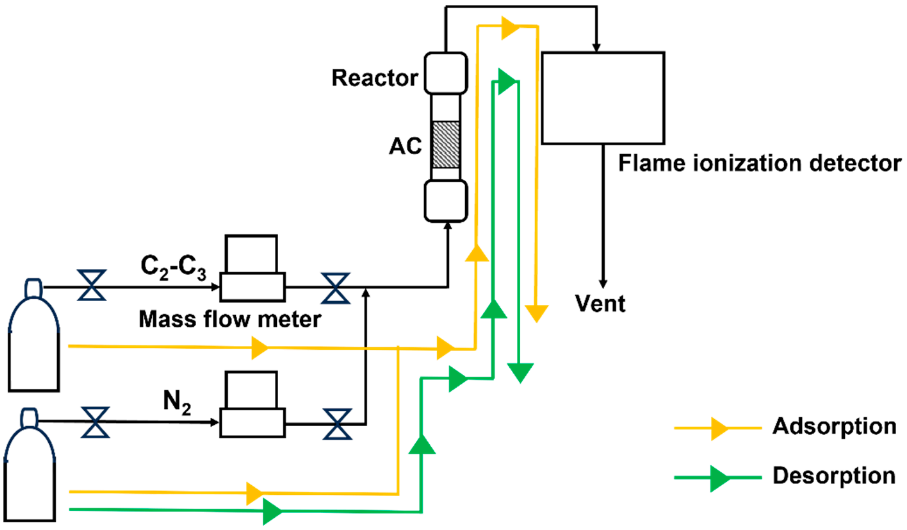

Figure S1. Schematic of the adsorption and desorption experiments.
